# Cardiovascular sex-differences: insights via physiology-based modeling and potential for noninvasive sensing via ballistocardiography

**DOI:** 10.3389/fcvm.2023.1215958

**Published:** 2023-10-06

**Authors:** Mohamed Zaid, Lorenzo Sala, Laurel Despins, David Heise, Mihail Popescu, Marjorie Skubic, Salman Ahmad, Craig A. Emter, Virginia H. Huxley, Giovanna Guidoboni

**Affiliations:** ^1^ Graduate School of Biomedical Science and Engineering, University of Maine, Orono, ME, United States; ^2^ Université Paris-Saclay, INRAE, MaIAGE, Jouy-en-Josas, France; ^3^ Sinclair School of Nursing, University of Missouri, Columbia, MO, United States; ^4^ Science, Technology & Mathematics, College of Arts and Sciences, Lincoln University, Jefferson City, MO, United States; ^5^ Health Management and Informatics, School of Medicine, University of Missouri, Columbia, MO, United States; ^6^ Electrical Engineering and Computer Science, College of Engineering, University of Missouri, Columbia, MO, United States; ^7^ Surgery, School of Medicine, University of Missouri, Columbia, MO, United States; ^8^ Biomedical Sciences, College of Veterinary Medicine, University of Missouri, Columbia, MO, United States; ^9^ Department of Medical Pharmacology and Physiology, School of Medicine, University of Missouri, Columbia, MO, United States; ^10^ National Center for Gender Physiology, University of Missouri, Columbia, MO, United States; ^11^ Electrical and Computer Engineering, Maine College of Engineering and Computing, University of Maine, Orono, ME, United States

**Keywords:** cardiovascular sex differences, noninvasive sensing, ballistocardiography, cardiovascular modeling, sex differeces, physiology-based modeling

## Abstract

In this study, anatomical and functional differences between men and women in their cardiovascular systems and how these differences manifest in blood circulation are theoretically and experimentally investigated. A validated mathematical model of the cardiovascular system is used as a virtual laboratory to simulate and compare multiple scenarios where parameters associated with sex differences are varied. Cardiovascular model parameters related with women’s faster heart rate, stronger ventricular contractility, and smaller blood vessels are used as inputs to quantify the impact (i) on the distribution of blood volume through the cardiovascular system, (ii) on the cardiovascular indexes describing the coupling between ventricles and arteries, and (iii) on the ballistocardiogram (BCG) signal. The model-predicted outputs are found to be consistent with published clinical data. Model simulations suggest that the balance between the contractile function of the left ventricle and the load opposed by the arterial circulation attains similar levels in females and males, but is achieved through different combinations of factors. Additionally, we examine the potential of using the BCG waveform, which is directly related to cardiovascular volumes, as a noninvasive method for monitoring cardiovascular function. Our findings provide valuable insights into the underlying mechanisms of cardiovascular sex differences and may help facilitate the development of effective noninvasive cardiovascular monitoring methods for early diagnosis and prevention of cardiovascular disease in both women and men.

## 1. Introduction

Women and men exhibit anatomical and functional differences in their cardiovascular systems. For example, women have smaller heart sizes, stronger ventricular contractility, smaller blood vessels, and smaller overall blood volume in the circulation (1–3). The manifestations of cardiovascular diseases also differ by sex. During a heart attack, men often present crushing chest pain, spreading pain in arms, nausea and cold sweat, whereas women mostly exhibit pain under the breastbone, abdominal pain, shortness of breath, nausea, and extreme fatigue (4, 5). In recent years, clinical studies have raised awareness of these differences along with the need to account for them to improve patient outcomes (6).

In this work, we contribute to this important area of research by investigating the effect of sex anatomical and functional differences on the circulation by means of a validated closed-loop mathematical model of the cardiovascular system (7–9). The model here is used as a virtual laboratory to simulate and compare multiple scenarios where parameters associated with sex differences are varied, such as ventricular contractility and arterial geometry. The model is also used to quantify the impact of sex-related parameter differences on the distribution of blood volume through the cardiovascular system. The model-predicted volumetric outputs are found to be consistent with published clinical data. Interestingly, the model indicates that the balance between the contractile function of the left ventricle (LV) and the load opposed by the arterial circulation, represented by the ventricular-arterial coupling (VAC) ratio, attains similar levels in females and males. This balance, however, is achieved through a different combination of factors. In females, the higher LV contractility is met by reduced arterial diameters, and this coupling ultimately leads to similar VAC ratios as in males.

Another interesting aspect of this work is that the mathematical model used to simulate cardiovascular sex differences also allows us to predict the shape of the ballistocardiogram (BCG) pertaining to the specific simulated scenario. As a matter of fact, the BCG signal is directly related to cardiovascular volumes. At each heartbeat, the blood ejected from the ventricles moves across the vascular compartments of the body which, as a consequence, host different amounts of blood at different instants along the cardiac cycle. The repetitive motion of blood volumes within the cardiovascular system results in the repetitive motion of the center of mass of the human body at each cardiac cycle, which is the motion captured by the BCG (10). Since the body motion is transmitted to the objects with which the body is in contact, the BCG offers a natural opportunity for noninvasive, unobtrusive monitoring of cardiovascular function. In the last decades, many devices for BCG sensing have been proposed, such as bed sensors (11, 12), weighing scales (13, 14), and accelerometers (15, 16).

A recent study showed that different BCG waveforms may be indicative of different cardiovascular baseline characteristics (15). Given the differences that the cardiovascular system exhibits in women and men, it is reasonable to conjecture that the baseline shape of the BCG signal may also be different depending on sex. Within this work, we test this conjecture by comparing the shape of BCG waveforms predicted by the mathematical model upon sex-related changes in cardiovascular parameters with BCG waveforms experimentally acquired on healthy males and females. Our study indicated that sex-related differences in arterial diameter and length are the major determinants of sex-related BCG differences, which manifest primarily through a decrease in amplitude and an earlier occurrence of the peaks, especially in the systolic phase.

The approach proposed in this work consists of utilizing mathematical modeling to interpret clinical and experimental data on the grounds of fundamental principles of cardiovascular physiology. This approach provides valuable insights on how different factors contribute to determine the healthy baseline conditions in women and men, which is a fundamental step towards a deeper understanding of sex differences in cardiovascular disease. Furthermore, we envision that the sex-related analysis of the BCG waveform will facilitate the effective design and implementation of noninvasive cardiovascular monitoring based on BCG sensing that will enable early diagnosis and prevent the worsening of cardiovascular disease in both women and men. The work is organized as follows. Sex-related cardiovascular differences observed in clinical studies are reviewed in Section 2. The main features of the mathematical model for the cardiovascular system are illustrated in Section 3, with particular emphasis on its inputs, outputs, and methods for BCG computing. The details of the experimental BCG acquisition are also provided in Section 3. The comparison between model predictions and clinical and experimental data is presented in Section 4, while conclusions and perspectives are outlined in Section 5.

## 2. Overview: sex-related cardiovascular differences

The cardiovascular system exhibits a similar structure in women and men, but its dimensions and functions are distinctly different depending on sex (5). The female heart size is, on average, one-fourth smaller than the male heart (3). Independently from the body size, women showed to have smaller ventricular chambers and smaller arterial diameter and length compared to men of the same age and race (1, 17). Table 1 summarizes the main findings related to differences in healthy hearts and arteries of males and females. The findings are also discussed below.

**Table 1:** Cardiovascular parameters for males and females.

Parameter	Male	Female	Reference
HR [beat/min]	74.3±8.9	79.1±8.2	(3)
Left ventricle			
EDV [mL]	168.4±27.2	124.0±27.1	(18)
ESV [mL]	78.6±20.3	53.5±11.9	(18)
SV [mL]	89.8±15.3	69.3±19.7	(18)
CO [L/min]	5.6±1.4	4.9±1.5	(18)
EF [%]	53.7±6.5	57.2±5.1	(18)
Ees [mm Hg/mL]	1.74	2.13	(22)
Ea [mm Hg/mL]	1.20	1.45	(22)
Ea/Ees ratio	0.69	0.68	(22)
Ed [mm Hg/mL]	0.063	0.081	(22)
Right ventricle			
EDV [mL]	157.9±47.5	132.5±46.7	(32)
ESV [mL]	80.9±43.3	65.2±44.0	(32)
SV [mL]	95.0±26.0	74.0±18.0	(33)
CO [L/min]	5.6±1.4	4.4±1.0	(33)
EF [%]	57.0±8.0	60.0±7.0	(33)
Ees [mm Hg/mL/m${}^{2}$]	0.7±0.2	0.8±0.2	(25)
Ea [mm Hg/mL/m${}^{2}$]	0.5±0.2	0.6±0.3	(25)
Ees/Ea ratio	1.4±0.4	1.7±0.9	(25)
Main arteries			
Diameter:			
Ascending aorta [cm]	3.4±0.4	3.1±0.5	(17)
Aortic arch [cm]	3.0±0.3	2.7±0.3	(17)
Thoracic aorta [cm]	2.5±0.3	2.3±0.3	(17)
Abdominal aorta [cm]	1.9±0.3	1.6±0.3	(17)
Carotid artery [cm]	0.65±0.10	0.61±0.10	(34)
Length:			
Ascending aorta [cm]	8.4±1.1	7.6±1.0	(17)
Aortic arch [cm]	3.7±0.8	3.2±0.7	(17)
Thoracic aorta [cm]	23.5±2.9	21.2±2.2	(17)
Abdominal aorta [cm]	14.4±2.2	12.8±2.0	(17)
Carotid artery [cm]	13.6±1.5	12.3±1.6	(30)
BCG amplitude [N]	5.56±1.74	3.56±1.06	(31)

Values are reported as mean ± standard deviation, when such information was available in the referenced articles.

HR, heart rate; EDV, end-diastolic volume; ESV, end-systolic volume; SV, stroke volume; CO, cardiac output; EF, ejection fraction; Ees, end-systolic elastance; Ea, arterial elastance; Ed, diastolic elastance; BCG, ballistocardiogram.

The Left Ventricle (LV) in women is typically smaller than in men, leading to lower end-diastolic volume (EDV) and end-systolic volume (ESV). The stroke volume (SV) is also smaller in women, being approximately 22.9% less than that in men (3, 18). The higher heart rate (HR) typically observed in women reduces the gap difference in the cardiac output (CO) to approximately 12.5% (1, 3, 18, 19). LV ejection fraction (EF), a meaningful indicator of ventricular efficiency, is found to be approximately 6.5% higher in women than in men (20). LV end-systolic elastance (Ees), an important marker of LV contractility, is also found to be higher in the female heart, with Ees being approximately 22.4% higher in women than in men (21). The increased value of Ees in females is accompanied by an increased value of diastolic elastance, Ed, and arterial elastance, Ea. Specifically, Ed and Ea are found to be approximately 28.6% and 20.8% higher in women than in men (22, 23). In a healthy heart, an increase in Ees is usually accompanied by an increase in Ea; this maintains the ventricular-arterial coupling (VAC) ratio (Ea/Ees ratio) within the healthy human range, approximately between 0.6 and 1.2, to ensure overall cardiovascular efficiency and performance (24). Interestingly, despite the many differences in anatomy and function, both the female and male hearts are characterized by similar VAC ratios very close to 0.7 (25).

The Right Ventricle (RV) in women is also characterized by smaller size and higher contractility compared to men (20, 26). Similarly to the LV, EDV and ESV in the RV are lower in females by 16.1% and 19.4%, respectively. The SV and the CO in the right ventricle are also lower in females. The female RV is characterized by values of EF, Ees, and Ea that are higher than those found in males by 5.2%, 14.3% and 20%, respectively (18, 22, 27). The end-diastolic elastance Ed, on the other hand, has not been found to be significantly different between the right ventricle of males and females (26). The RV-Pulmonary Arterial (RV-PA) coupling estimated by RV Ees/Ea ratio is considered to be an indicator of RV efficiency. Its healthy range falls between 1.0 and 2.0 for healthy individuals; in males and females it is reported at 1.7 and 1.4, respectively (25, 28).

The Main Arteries also present important differences among women and men. Vessel diameters and lengths are usually smaller in females compared to males (1, 29). Diameter and length of the arterial root are, on average, approximately 10% smaller in females than in males. This difference, however, seems to vary along the aortic segments. Specifically, the female-male differences in the diameters of the ascending aorta, the aortic arch, the thoracic aorta, and the abdominal aorta are found to amount approximately to 8.8%, 10%, 8.0%, and 15.8%, respectively (17). The diameter of the carotid artery is approximately 6.2% smaller in females compared to males (30). Similarly, arteries are typically shorter in women, for whom the length of ascending aorta, the aortic arch, the thoracic aorta, and the abdominal aorta are approximately 9.5%, 13.5%, 9.8% and 11.1% smaller than in men (17). The carotid length is reported to be 9.6% smaller in women (30).

It is reasonable to assume that the female-male differences in the cardiovascular system will also manifest in BCG signals. We recall that the BCG waveform results from the motion of the center of mass of the human body as the blood volume redistributes within different vascular compartments at each heartbeat (10). Thus, anatomic and functional differences in the cardiovascular system may lead to different patterns in blood volume distribution, which could be picked up by the BCG. Indeed, it has been observed that the mean BCG amplitude in females is lower than in males by nearly 36% (3.56 N vs. 5.56 N) (31).

## 3. Methods

This study utilizes a validated mathematical model for the cardiovascular system to predict and quantify how the distribution of blood volume within the body is impacted by differences in specific cardiac and vascular parameters associated with males and females (Section 2). The cardiovascular model leverages the analogy between the flow of a fluid in a hydraulic network and the flow of current in an electric circuit and translates fundamental principles of cardiovascular physiology into mathematical equations, a comprehensive description of the cardiovascular model, with full details, is provided in (7). Here we focus on describing which parameters are used as model inputs (Section 3.1), which quantities are computed as model outputs (Section 3.3), and which numerical strategies are used to solve the equations (Section 3.4). The model-predicted BCG waveforms are compared with experimental BCG waveforms acquired on human subjects by means of an accelerometer placed on a suspended bed. The details of the experimental BCG acquisition are given in Section 3.5.

### 3.1. Cardiovascular model inputs

**Figure 1:**
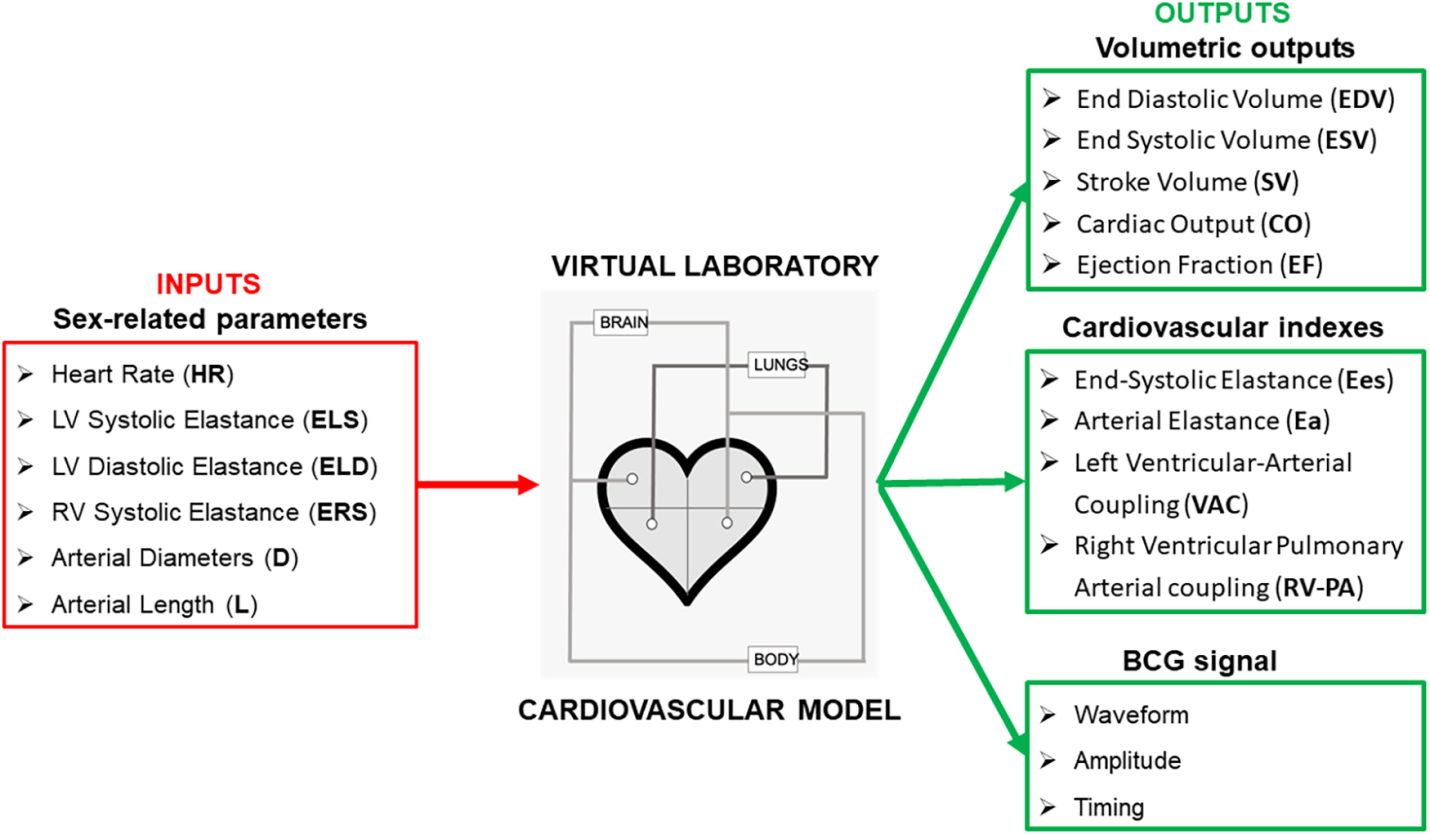
Selected parameters that have been found to be significantly different among women and men are used as inputs for the cardiovascular model proposed in Guidoboni et al. (7) to estimate volumetric outputs of the left and right ventricles, along with cardiovascular indeces and the ballistocardiogram (BCG) waveform.

The cardiovascular model schematized in the central panel of Figure 1 is described in detail in Guidoboni et al. (7). The pumping action of the ventricles, represented by a voltage source and a time-varying capacitor connected in series, is driving the blood flow through the systemic and pulmonary circulations. Resistances and inductances along the circuit represent viscous and inertial effects, respectively, of the blood flowing through. Capacitances capture the compliance of blood vessels, which can deform and accommodate different levels of blood volume throughout the cardiac cycle.

In the following, we illustrate how specific parameters exhibiting sex-related differences are accounted for in the model, referring the interested reader to (7) for the full model details.

∙ *Heart rate.* The HR can be used as a direct input of the model through the parameters that define the activation functions $a_{L}(t)$ and $a_{R}(t)$ for the left and right ventricles, respectively (see Eq. (6g) in (7)). The functions $a_{L}(t)$ and $a_{R}(t)$ are periodic and their period $T_{c}$ can be determined from HR as
  (1) $$T_{c}=\frac{60}{\text{HR}}\;\text{[ s] }$$
  where HR is measured in beats/min. In order to account for the higher HR that is typically observed in females (see Section 2), we have assumed the HR in our female model to be 5% higher than in our male model. This led us to adopt the values of 75 beats/min and 78.75 beats/min for the HR in our male and female models, respectively.
∙ *Ventricular properties.* Each ventricle in the cardiovascular model is described via a pressure generator capturing the isovolumic contraction, connected in series with a time-varying elastance accounting for the tension-length curve of activated fibers and the ventricular geometry. In particular, the elastances for the left and right ventricles, denoted by $E_{L}(t)$ and $E_{R}(t)$, respectively, are related to the activation functions $a_{L}(t)$ and $a_{R}(t)$ via the following constitutive equations:
  (2) $$E_{L}(t)=ELD+ELS\;a_{L}(t),\;E_{R}(t)=ERD+ERS\;a_{R}(t)$$
  where ELD, ELS, ERD and ERS are parameters that can be set to different values for women and men. Specifically, we have assumed ELS, ERS, and ELD to be higher in the female model (see Table 2). Conversely, ERD was assumed to be the same for both the female and male models, since no significant differences were reported in the literature for the values of Ed in the right ventricle.
∙ *Arterial diameter and length.* Diameter and length of the main arteries are a direct input for the cardiovascular model, as they enter explicitly in the formulas to compute the vessel resistance R, inductance L, and capacitance C reported below for ease of reference:
  (3) $$R=\frac{128l\eta }{\pi d^{4}},\;L=\frac{4\rho_{b}l}{\pi d^{2}},\;C=\frac{3l\pi d^{2}h(d+2h{)}^{2}}{16E(d+h)}.$$
  In these formulas, d and l represent the vessel diameter and length, respectively, whereas η and $\rho_{b}$ represent the blood viscosity and density, and h and E represent the thickness and the Young modulus of the vessel wall, respectively. In order to account for the smaller vessel diameters and lengths that are typically observed in females (see Section 2), we have assumed d and l for all major arteries to be 10% smaller than in our male model.

**Table 2:** Input parameter values for the 8 different versions of the cardiovascular model considered in this work, from the original parameters employed in (7), to represent the *idealized male* to the parameters used to describe the *idealized female*.

	Model versions							
	Idealized male	HR	ELS	ELD	ERS	Arterial diameter	Arterial length	Idealized female
Heart Rate [beat/min]	75	79	75	75	75	75	75	**78.75**
ELS [mmHg cm${}^{-3}$ s${}^{-1}$]	1.375	1.375	1.581	1.375	1.375	1.375	1.375	1.581
ELD [mmHg cm${}^{-3}$]	0.04	0.04	0.04	0.05	0.04	0.04	0.04	0.05
ERS [mmHg cm${}^{-3}$ s${}^{-1}$]	0.23	0.23	0.23	0.23	0.27	0.23	0.23	0.27
Arterial diameter [cm]								
Ascending aorta	1.44	1.44	1.44	1.44	1.44	1.30	1.44	1.30
Aortic arch	1.14	1.14	1.14	1.14	1.14	1.03	1.14	1.03
Thoracic aorta	0.96	0.96	0.96	0.96	0.96	0.86	0.96	0.86
Abdominal aorta	0.85	0.85	0.85	0.85	0.85	0.77	0.85	0.77
Iliac artery	0.52	0.52	0.52	0.52	0.52	0.47	0.52	0.47
Carotid artery	0.39	0.39	0.39	0.39	0.39	0.35	0.39	0.35
Arterial length [cm]								
Ascending aorta	4.0	4.0	4.0	4.0	4.0	4.0	3.6	3.6
Aortic arch	5.9	5.9	5.9	5.9	5.9	5.9	5.31	5.31
Thoracic aorta	15.6	15.6	15.6	15.6	15.6	15.6	14.04	14.04
Abdominal aorta	15.9	15.9	15.9	15.9	15.9	15.9	14.31	14.31
Iliac artery	5.8	5.8	5.8	5.8	5.8	5.8	5.22	5.22
Carotid artery	20.8	20.8	20.8	20.8	20.8	20.8	18.72	18.72

Boldface fonts highlight the changes from the original model values retrieved from (7).

### 3.2. Cardiovascular model versions

In order to assess the impact that the change in each of the aforementioned inputs has on the distribution of blood volume and pressure throughout the cardiovascular system, we proceed by considering the following different versions of the model:

∙ *Idealized male model:* in this version, henceforth referred to as *male model* for simplicity, all the values of the model parameters are the same as those reported in (7);
∙ *HR model:* in this version, all model parameters are the same as for the male model except for HR, which is assumed to be 5% higher than what was reported in (7);
∙ *ELS model:* in this version, all model parameters are the same as for the male model except for ELS, which is assumed to be 15% higher than what was reported in (7);
∙ *ELD model:* in this version, all model parameters are the same as for the male model except for ELD, which is assumed to be 30% higher than what was reported in (7);
∙ *ERS model:* in this version, all model parameters are the same as for the male model except for ERS, which is assumed to be 15% higher than what was reported in (7);
∙ *Arterial diameter model:* in this version, all model parameters are the same as for the male model except for the diameters of the major arteries, which are assumed to be 10% smaller than what was reported in (7);
∙ *Arterial length model:* in this version, all model parameters are the same as for the male model except for the lengths of the major arteries, which are assumed to be 10% smaller than what reported in (7);
∙ *Idealized female model:* in this version, henceforth referred to as *female model* for simplicity, the changes in HR, arterial diameter and length, ELS, ELD and ERS listed above are implemented simultaneously.

By comparing the cardiovascular outputs (see Section 3.3) obtained for each model version listed above, we will be able to study the effect of changing the value of one parameter at a time versus changing them all together. The summary of the parameter values pertaining to each version are summarized in Table 3. Boldface fonts have been utilized to emphasize the values of those parameters that differ from the rest. The values of the model parameters not reported explicitly in Table 2 are assumed to be the same as those in (7).

**Table 3:** Details of the subjects recruited for the synchronous acquisition of ECG and BCG signals.

Subject ID	Sex	Age	Height (cm)	Weight (kg)
1M	Male	25	189.0	72.6
2M	Male	23	179.8	54.0
3M	Male	32	180.0	70.0
1F	Female	26	164.6	47.6
2F	Female	28	165.0	49.9
3F	Female	29	163.0	50.1

### 3.3. Cardiovascular model output

For a given set of parameters, the outputs of the cardiovascular model are quantities computed from the solution of the system of nonlinear ordinary differential equations describing the model (see Appendix of (7)). In this study we will be focusing on three main types of outputs: volumetric outputs, cardiovascular indexes, and BCG waveform.

∙ *Volumetric outputs.* The model-predicted EDV and ESV are computed as the maximum and minimum values, respectively, of the simulated volume waveforms for the left and right ventricles. From these values, we compute SV=EDV−ESV, CO=HR×SV, and EF=SV/EDV.
∙ *Cardiovascular indexes.* The model-predicted end-systolic pressure (ESP) is computed as the maximum pressure value simulated for the left and right ventricles. From these values and the volumetric outputs, we compute Ea=ESP/SV, Ees=ESP/ESV, VAC=Ea/Ees, and RV-PA=Ees / Ea.
∙ *BCG waveform.* The model-predicted volume waveforms at the various nodes of the model provide the time-dynamics of how blood volume redistributes throughout the cardiovascular system. Using basic physics principles (7, 10), this information can be used to calculate the force associated with the motion of the center of mass of the human body, which gives rise to the BCG waveform.

### 3.4. Model implementation and solution

The cardiovascular model has been implemented using OpenModelica (35), an open-source modelica-based modeling and simulation environment. The mathematical equations representing the system have been solved using a differential algebraic system solver, DASSL, with time step of 0.001 s and tolerance of $10^{-6}$, as in (7). Exploiting the library PyFMI, a Python script has been written to call a functional mockup unit (FMU) generated by OpenModelica that can solve the cardiovascular model for specified input values. In order to ensure that the solution reaches a periodic behavior, the system is solved over a time interval of 8 cardiac cycles. The solution segment corresponding to the last cardiac cycle is then considered for analysis. Post-processing of results has also been implemented in Python.

### 3.5. Experimental data acquisition

Six subjects were recruited for this study. Sex, age, height, and weight are reported in Table 3. Data collection was performed in a controlled laboratory environment, where subjects were asked to lie on the suspended bed described previously in (7). ECG and BCG were synchronously recorded by a three-lead configuration and by a Kionix accelerometer with 1000 mV/g sensitivity placed on the suspended bed frame, respectively. An ADInstrument PowerLab 16/35 data acquisition system was used to collect the signals synchronously. ECG and BCG signals were both filtered via a 6th-order Butterworth bandpass filter to remove the high-frequency noise and the low-frequency respiration movement. ECG and BCG signals were filtered with a cut-off frequency of [0.7–40] and [1.25–15] Hz, respectively (15). R peaks in the ECG signal were used to segment the BCG signal at each cardiac cycle. For each subject, the mean BCG wave of the bundle of segmented waveforms was used as a pattern.

## 4. Results

The outputs of the cardiovascular model obtained for the various versions illustrated in Section 3.2 are compared in terms of volumetric outputs (Section 4.1), cardiovascular indexes (Section 4.2), and BCG waveforms (Section 4.3). BCG waveforms estimated from the model are then compared with the BCG signals obtained experimentally (Section 4.3). We recall that, starting from the idealized male model version based on the model parameters reported in (7), we vary specific parameter values individually (i.e. HR, ELS, ELD, ERS, arterial diameter and length), thereby yielding six additional model versions. All changes are simultaneously incorporated into a single version called idealized female model version. Since the specific values of the modified parameters reported in Table 2 may vary from person to person, we also tested the cases in which such values were altered by ±5%.

### 4.1. Comparison of volumetric outputs

**Figure 2:**
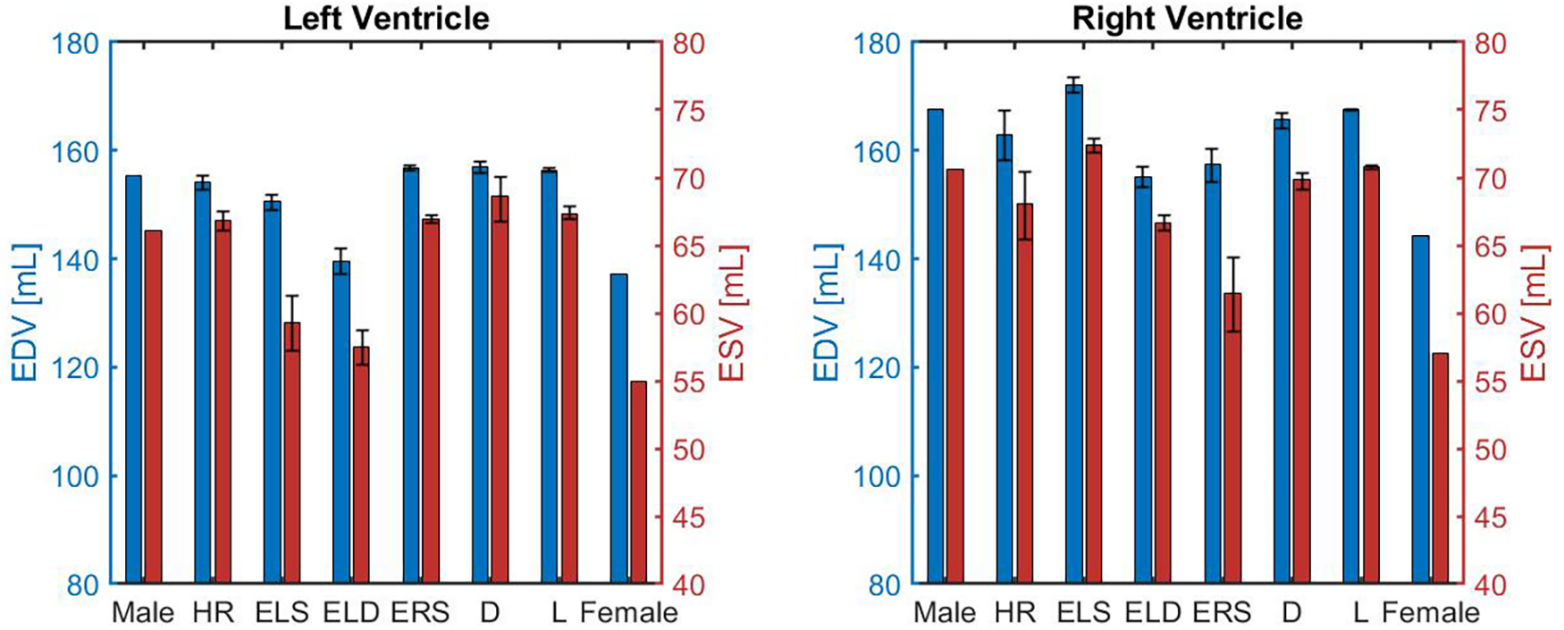
Comparison of left and right ventricular end-diastolic volume (EDV) and end-systolic volume (ESV) obtained for different model versions, namely the idealized male model (Male), HR model (HR), ELS model (ELS), ELD model (ELD), ERS model (ERS), Arterial diameter model (D), Arterial length model (L), and the idealized female model (Female) described in Section 3.2.

Figure 2 shows the EDV (shown as blue bars) and ESV (shown as red bars) for the LV (left panel) and the RV (right panel). The error bars indicate the change in outputs due to ±5% alteration in the parameter value that characterizes the corresponding model. The EDV and ESV obtained for the LV in the female model are 137.2 and 54.9 mL, respectively. These values are 11.7% and 10.1% lower than those obtained for the male model, where EDV and ESV result to be 155.4 and 66.1 mL, respectively. These volume values are within the ranges reported in clinical studies, which are reported alongside the model predictions in Table 4 for ease of comparison. The barplots suggest that the increase in ELD and ELS contribute the most to this volumetric difference. These results seem reasonable since a higher ELD value corresponds to a reduction in ventricular relaxation that limits the filling phase, whereas a higher ELS value corresponds to a stronger contractility that reduces the volume of blood remaining in the LV after each contraction.

**Table 4:** Comparison between model predictions and clinical data for cardiovascular markers in males and females.

Parameter	Male		Female	
	Model prediction	Clinical data	Model prediction	Clinical data
Left ventricle				
EDV [mL]	155.4	168.4±27.2	137.2	124.0±27.1
ESV [mL]	66.1	78.6±20.3	54.9	53.5±11.9
SV [mL]	89.2	89.8±15.3	82.3	69.3±19.7
CO [L/min]	6.7	5.6±1.4	6.4	4.9±1.5
EF [%]	57.4	53.7±6.5	60.0	57.2±5.1
Ees [mm Hg/mL]	2.08	1.74	2.54	2.13
Ea [mm Hg/mL]	1.54	1.20	1.7	1.45
Ea/Ees ratio	0.74	0.69	0.67	0.68
Right ventricle				
EDV [mL]	167.7	157.9±47.5	144.3	132.5±46.7
ESV [mL]	70.6	80.9±43.3	57.0	65.2±44.0
SV [mL]	97.1	95.0±26.0	87.3	74.0±18.0
CO [L/min]	7.3	5.6±1.4	6.9	4.4±1.0
EF [%]	57.9	57.0±8.0	60.5	60.0±7.0
Ees [mm Hg/mL]	0.56	0.7±0.2	0.68	0.8±0.2
Ea [mm Hg/mL]	0.41	0.5±0.2	0.44	0.6±0.3
Ees/Ea ratio	1.38	1.4±0.4	1.53	1.7±0.9
BCG amplitude [$10^{5}$ Dyne]	1.93	2.26±0.48	1.43	1.21±0.07

The reference for the clinical data are the same as those reported in Table 1.

HR, heart rate; EDV, end-diastolic volume; ESV, end-systolic volume; SV, stroke volume; CO, cardiac output; EF, ejection fraction; Ees, end-systolic elastance; Ea, arterial elastance; Ed, diastolic elastance; BCG, ballistocardiogram.

Similarly to the LV, the blood volumes predicted by the model in the female RV are lower than in males. The model-predicted EDV and ESV in the RV of the female model are 144.3 and 57.0 mL compared to 167.7 and 70.6 mL obtained for males. The smaller blood volumes predicted for both ventricles of the female model are consistent with the smaller ventricular size observed in women (18).

**Figure 3:**
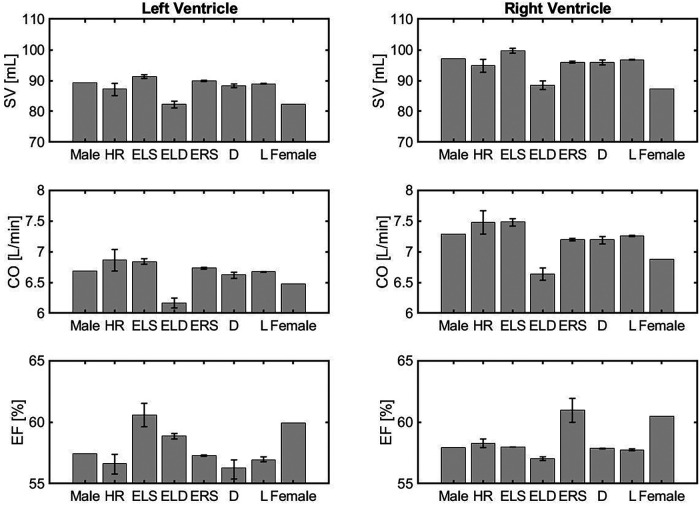
Comparison of volumetric outputs obtained for different model versions, namely the idealized male model (Male), HR model (HR), ELS model (ELS), ELD model (ELD), ERS model (ERS), Arterial diameter model (D), Arterial length model (L), and the idealized female model (Female) described in Section 3.2.

Figure 3 is organized in three rows, reporting the results for stroke volume (SV), cardiac output (CO) and ejection fraction (EF), and two columns, corresponding to the left and right ventricles (LV, RV). The model simulations predict lower SV for both the LV and the RV in the female model when compared to the male model. The percent difference is 7.7% in the LV and 10% in the RV. Interestingly, when compared to the male model, the SV is higher in the ELS model, due to increased contractility, and lower in the ELD model, due to higher diastolic stiffness. The latter effect seems to be predominant since, overall, the SV predicted for the female model is lower than in the male model. Interestingly, the increase in HR compensates for the SV reduction leading to a simulated CO for the female model that is only 4.5% smaller than in the male model for the LV and 5.5% for the RV. Furthermore, the model predicted EF for the female model is higher than the value obtained for the male model in both ventricles. The barplots show that this is mainly due to the increase in ELS and ERS, which represent stronger LV and RV ventricular contractions, respectively. These results are consistent with the trends exibited by the clinical data reported in Table 4.

### 4.2. Comparison of cardiovascular indexes

**Figure 4:**
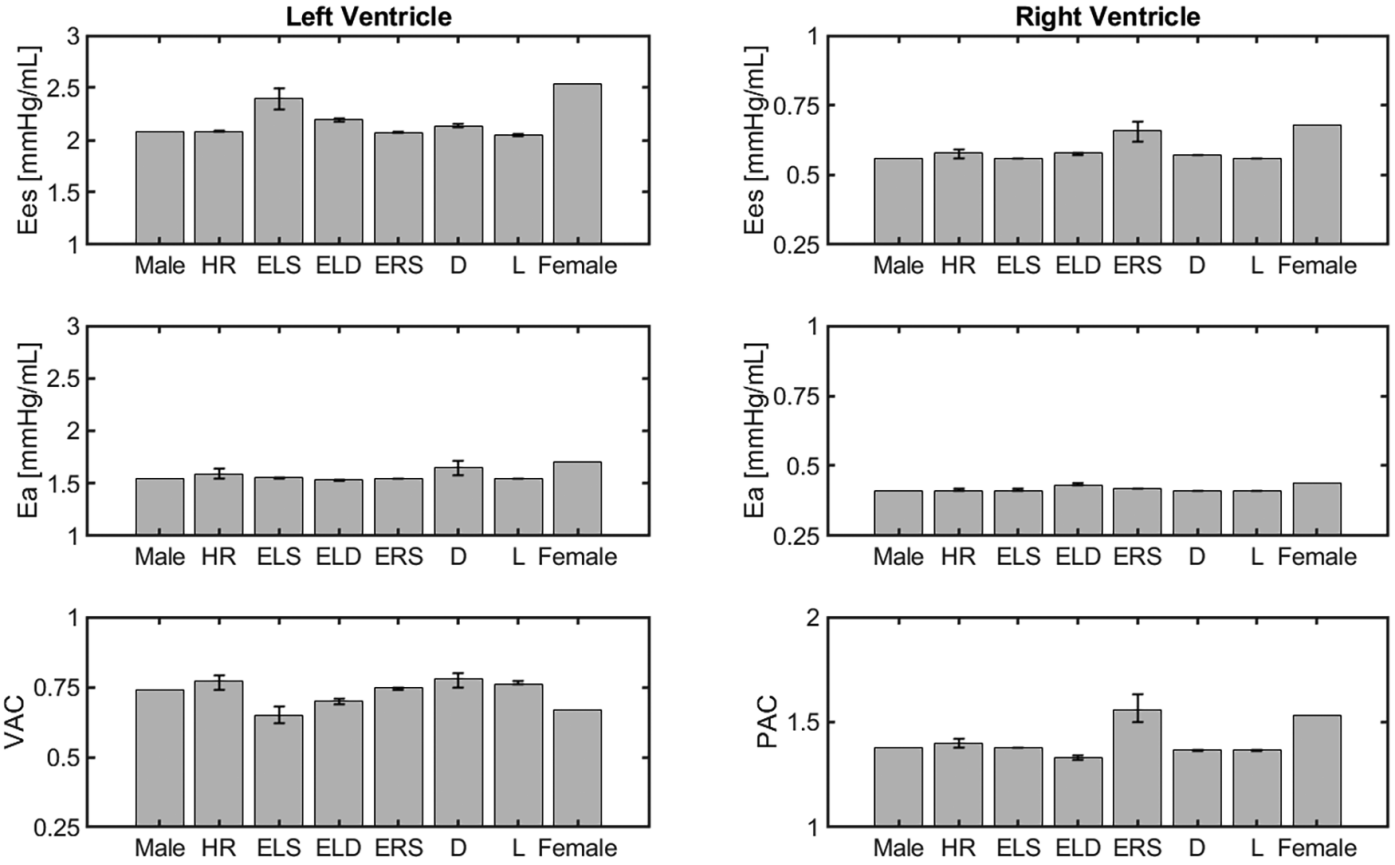
Comparison of cardiovascular indexes obtained for different model versions, namely the idealized male model, HR model, ELS, model, ELD model, ERS model, Arterial diameter model, Arterial length model, and the idealized female model described in Section 3.2.

Cardiovascular indexes obtained with the different versions of the cardiovascular model are reported as barplots in Figure 4. The panels in the figure are organized in 3 rows, reporting the results of Ees, Ea and their ratio for the left and right ventricles. The error bars indicate the change in outputs due to ±5% alteration in the parameter value that characterizes the corresponding model.

The model simulations predict higher Ees values in the female model compared to the male model. This is mainly due to an increase in ventricular contractility, represented by ELS in the LV and ERS in the RV. This is consistent with the clinical findings, as summarized in Table 4.

The model predicted Ea values for both the LV and RV are slightly higher in the female model than in the male model. As a result, the VAC ratios obtained for the female and male models are quite similar, equal to 0.67 and 0.74 respectively, and within the healthy human range [0.6–1.2] reported in the literature (25). The simulated RV-PA coupling (PAC) in the female and male models result to be 1.53 and 1.38, respectively, which are also within the reported optimal coupling range of [1.0–2.0] (28).

### 4.3. Comparison of predicted BCG signal

**Figure 5:**
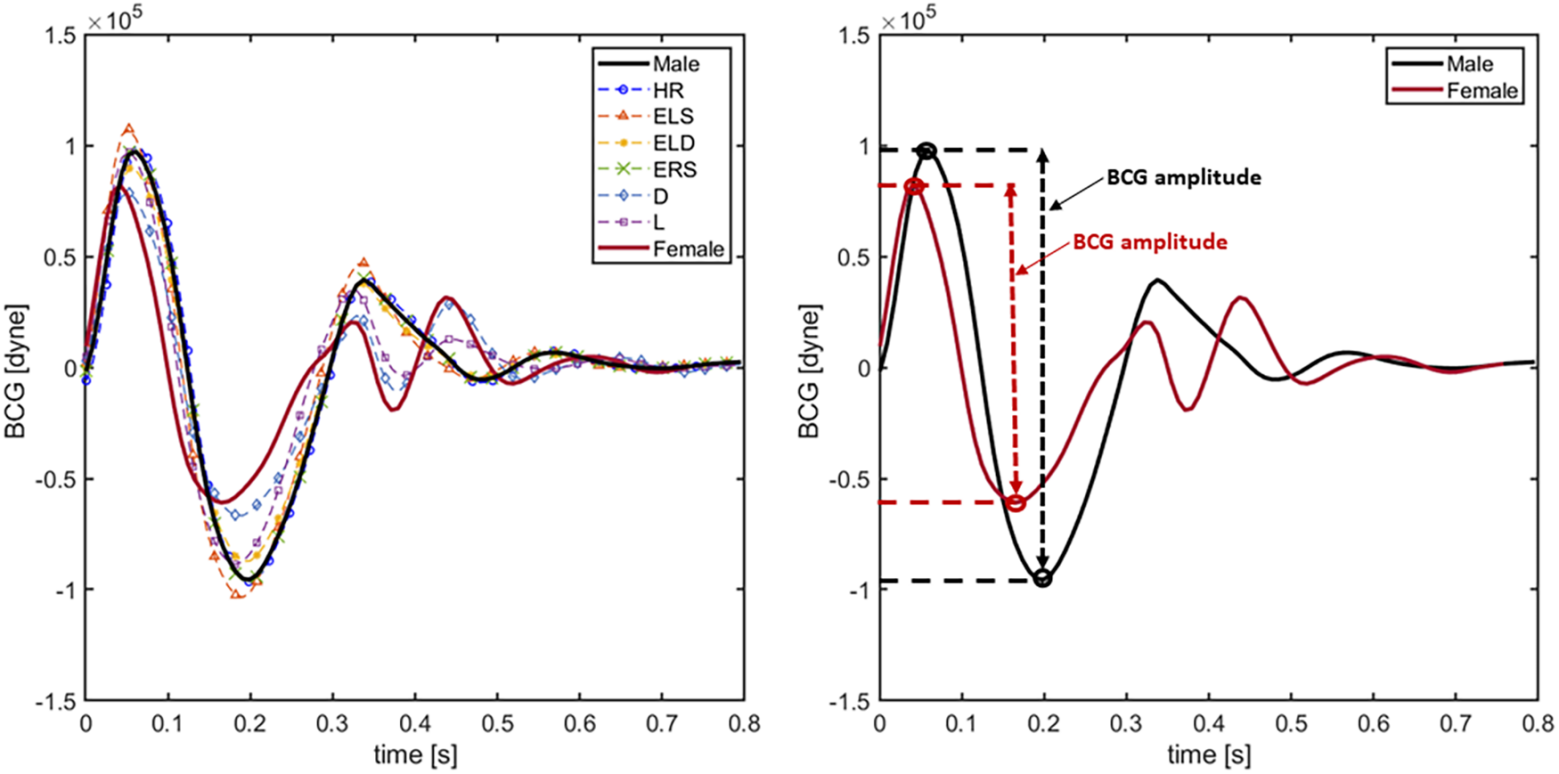
BCG waveforms obtained simulating the 8 different models presented in Section 3.1. In particular solid black line indicates the result employing the original *idealized male* model. Solid red line is obtained using the *idealized female* model. Blue, orange, yellow, violet, green and cyan dashed lines represent the BCG waveforms using the HR, ELS, ERS, arterial diameter (D) and arterial length (L) models, respectively.

**Figure 6:**
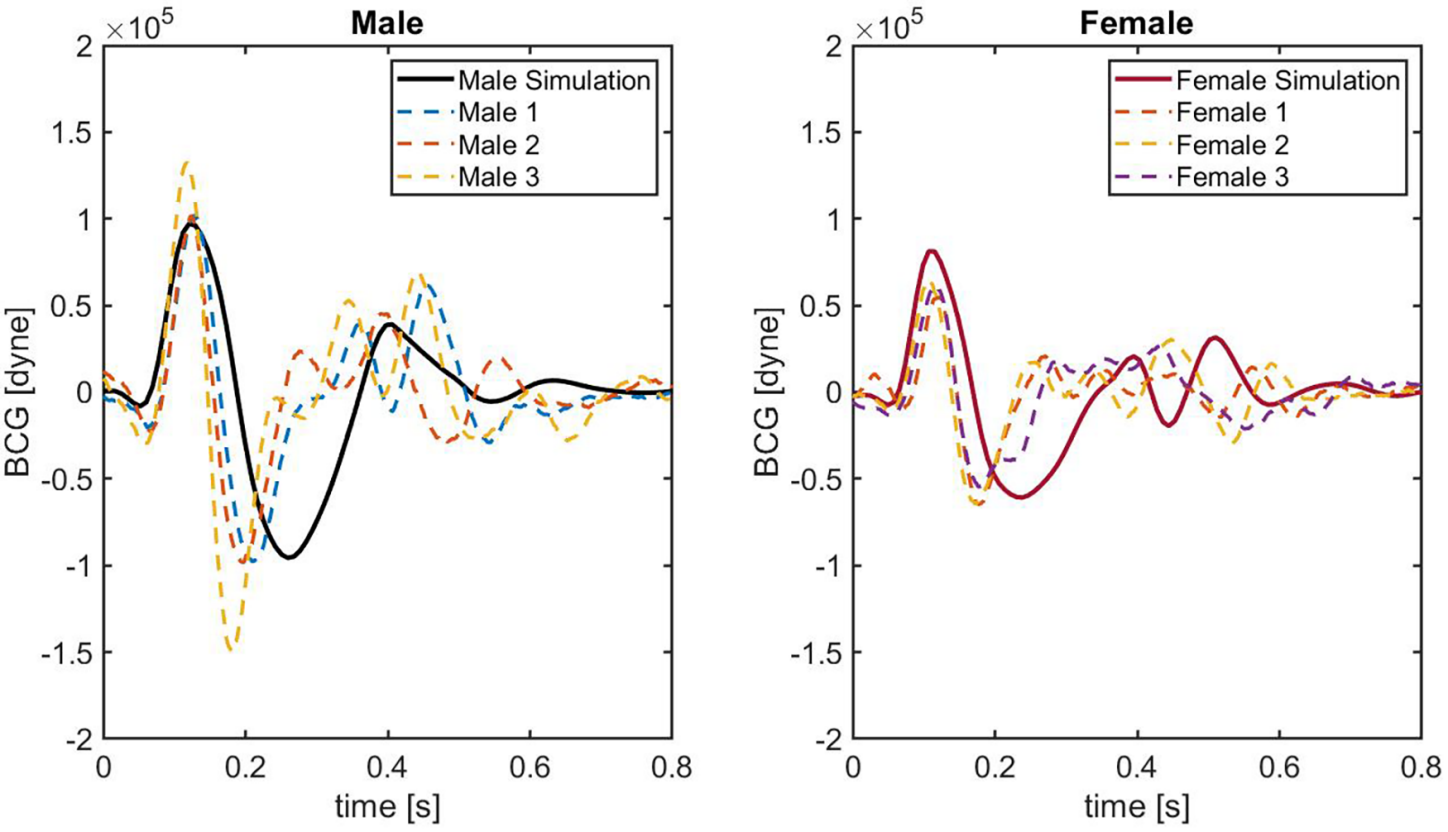
BCG waveforms obtained on 6 different healthy subjects. Solid black line indicates the result employing the original *idealized male* model. Solid red line is obtained using the *idealized female* model. Colored dashed lines represent the BCG waveforms acquired on each subject.

The BCG waveforms reconstructed virtually with the 8 different models presented in Section 3.2 are displayed in Figure 5. Specifically, *idealized female* and *idealized male* model simulations are highlighted in red and black solid lines, respectively, whereas the other cases are reported in dashed lines. Model simulations predict that an increase in ELS (red dashed line with triangles) enhances and anticipates the peak of the systolic phase in the BCG waveform. This result is consistent with the findings reported in (9), where the associations between changes in ventricular contractility and changes in BCG amplitude and timing were established on a preclinical swine model using induced myocardial infarction. Simulations also predict that a reduction in arterial diameter (blue dashed line with diamonds) decreases and anticipates the peak of the systolic phase in the BCG waveform. Moreover, the effect of the arterial diameter reduction is reflected in the post-systolic phase of the BCG, where the shape of the waveform differs from the male model with the presence of double peaks between 0.3 and 0.5 s. This result is consistent with the findings of Inan et al. where authors hypothesize that the second peak in the BCG waveform could be related to the mechanical resonance of the vasculature (31). Indeed, similar simulation results in the post-systolic phase can be observed when the arterial length is reduced (purple dashed line with squares). This behavior in the BCG waveform might be traced back to the fact that smaller and shorter tubes induce faster wave propagation. On the RV side, an increase in the value of ERS (violet dashed line in Figure 6) does not seem to influence the BCG waveform. This interesting finding suggests that the BCG signal may not be strongly influenced by RV function. When the BCG waveform obtained for the various model versions is compared with that obtained for the *idealized female* model, it appears that the reduction of arterial diameter is the dominant feature affecting sex-related BCG changes. Indeed, female BCG predicted amplitude has been found to be 25.9% smaller than in the proposed *idealized male* model. This important outcome is in line with the results of (31, 36).

Figure 6 shows the comparison between model-predicted and experimentally-measured BCG waveforms. In the left panel, the BCG waveform for the idealized male model (black solid curve) is compared with the experimental waveforms of three male subjects (dashed curves). Similarly, in the right panel the BCG waveform for the idealized female model (red solid curve) is compared with the experimental waveforms of three female subjects (dashed curves). Notably, in the systolic phase between 0.0 and 0.3 s, the agreement between the model-predicted BCG for male and female and the measured BCG curves is quite satisfactory. The similarity in the systolic peaks is clearly detectable, both in terms of timing and amplitude. While comparable in terms of amplitude, shape of the BCG waveform in the post-systolic phase reported experimentally is more complex than the one predicted by the model. This result is not surprising, since capturing the features of the BCG in the post-systolic phase remains challenging both experimentally and theoretically (7, 10), and it motivates further research in this direction.

## 5. Conclusions and future perspective

This study provides novel insights on the effect of sex-related anatomical and functional differences on blood circulation. Theoretical predictions based on a mechanism-driven cardiovascular model were used to interpret clinical and experimental data and describe how different factors contribute to determine the healthy baseline conditions in women and men. This is a fundamental step towards a deeper understanding of sex differences in cardiovascular disease. Our results indicate that the balance between LV contractile function and arterial impedance is achieved differently in females and males, with higher LV contractility in females being met by smaller arterial diameters. This difference in cardiovascular balance is captured by the BCG waveform, where the reduced arterial diameters lead to smaller amplitudes and earlier timing of the BCG peaks. These insights help deepen our understanding of sex-related baseline differences in cardiovascular function and could enable better tailoring of therapeutic and monitoring approaches to cardiovascular disease in women and men. Thanks to its noninvasiveness and strict relationship with blood volume distribution, sex-informed BCG sensing could be used to monitor cardiovascular changes in many different situations, spanning from optimizing physical exercise (37, 38) to detecting risks during pregnancy (39, 40).

Noninvasive cardiovascular monitoring based on BCG waveforms can provide advantages with respect to accessibility and cost-effectiveness when compared to other techniques such as echocardiography, making BCG-based devices suitable for routine monitoring in home and ambulatory settings. Routine monitoring could potentially lead to early diagnosis of cardiovascular complications, facilitating timely interventions and reducing the burden of cardiovascular diseases. While BCG waveforms hold great promise, a critical challenge for widespread clinical use is the standardization of BCG waveform acquisition. To-date, several devices and methods have been used to measure the BCG in various settings, and these are not always comparable. Therefore, the great challenge is to standardize BCG measurement to ensure that data collected from different sources can be consistent and comparable.

The results presented in this work should be contextualized within the limitations of the methods utilized to obtain them. The model simulations have been compared with published values of cardiac volumes and related parameters. These values, however, should be considered more as general indicators than as exact numbers since they may vary depending on the particular subjects under consideration. The cardiovascular model considered here does not include a detailed description of the atria, which could affect the ability to capture post-systolic events in the distribution of blood volumes and, consequently, in the BCG waveform. Furthermore, the comparison between experimentally-measured and model-predicted BCG waveforms could benefit from a larger pool of subjects. A larger cohort spanning across ages would better facilitate insights into how aging may affect cardiovascular function in women and men. These are very important directions of research that could be explored by extending the approach presented here.

## Data Availability

The raw data supporting the conclusions of this article will be made available by the authors upon request.
